# Vitamin D_3_ suppresses intestinal epithelial stemness via ER stress induction in intestinal organoids

**DOI:** 10.1186/s13287-021-02361-2

**Published:** 2021-05-13

**Authors:** Panida Sittipo, Hyun Kyu Kim, Jaeseok Han, Man Ryul Lee, Yun Kyung Lee

**Affiliations:** Department of Integrated Biomedical Science, Soonchunhyang Institute of Medi-Bio Science, Soonchunhyang University, Cheonan, 31151 Republic of Korea

**Keywords:** Intestinal epithelial cells (IECs), Intestinal organoids, Vitamin D_3_, Endoplasmic reticulum (ER) stress

## Abstract

**Background:**

Vitamin D_3_ is important for normal function of the intestinal epithelial cells (IECs). In this study, we aimed to investigate the effects of vitamin D_3_ on the differentiation, stemness, and viability of healthy IECs in intestinal organoids.

**Methods:**

Intestinal organoids derived from mouse small intestine were treated with vitamin D_3_, and the effects on intestinal stemness and differentiation were evaluated using real-time PCR and immunofluorescence staining of the distinct lineage markers. Cell viability was analyzed using viability and apoptosis assays.

**Results:**

Vitamin D_3_ enhanced IEC differentiation into the distinct lineages of specialized IECs, including Paneth, goblet, and enteroendocrine cells and absorptive enterocytes. Decreased expression levels of leucine-rich repeat-containing G-protein-coupled receptor 5 (LGR5) and the presence of several LGR5-green fluorescent protein (GFP)-positive cells were observed in vitamin D_3_-treated organoids derived from LGR5-GFP mice. The formation of the crypt-villus structure was also inhibited by vitamin D_3_, suggesting that vitamin D_3_ suppresses intestinal cell stemness. Furthermore, the expression levels of unfolded protein response genes, C/EBP homologous protein (CHOP), and activating transcription factor 6 (ATF6) were upregulated in vitamin D_3_-treated organoids. Moreover, vitamin D_3_ promoted apoptotic cell death in intestinal cells, which may be associated with the decrease in intestinal stemness. LGR5 gene expression, ISC number, and apoptotic cell death were partially recovered in the presence of the ER stress inhibitor tauroursodeoxycholic acid (TUDCA), suggesting that intestinal stemness suppression and intestinal apoptosis occurred via ER stress activation.

**Conclusions:**

Our study provides important insights into the effects of vitamin D_3_ on the induction of IEC differentiation and apoptotic cell death, and inhibition of intestinal stemness accompanied by ER stress augmentation.

**Supplementary Information:**

The online version contains supplementary material available at 10.1186/s13287-021-02361-2.

## Background

1,25-Dihydroxyvitamin D_3_ (vitamin D_3_) is an active form of vitamin D [1, 2]. Vitamin D_3_ has a broad range of biological activities and is primarily responsible for intestinal absorption of calcium and phosphorus [3, 4]. In addition, vitamin D_3_ plays a role in maintaining intestinal barrier function, which prevents bacterial translocation and ensures that appropriate inflammatory responses take place by regulating tight junction gene expression in the intestine [5, 6]. Furthermore, vitamin D_3_ contributes to detoxification, protection against infection, and cancer suppression [7, 8]. Vitamin D_3_ deficiency is associated with many intestinal diseases, such as inflammatory bowel diseases, short bowel syndrome, and pancreatitis [8, 9]. Moreover, supplementation with high-dose vitamin D_3_ was found to increase colitis susceptibility in a dextran sulfate sodium-induced colitis mouse model [10]. However, direct evidence showing the effect of vitamin D_3_ on intestinal epithelial cells (IECs) in the small intestine under homeostasis is limited. Therefore, it is necessary to study the dose-dependent effects of vitamin D_3_ on IECs in the small intestine.

IECs form a single layer epithelium, which functions as a physical barrier and supports host health [11]. IECs comprise various specific cell lineages originating from intestinal stem cells (ISCs). ISCs undergo proliferation and differentiation into secretory cells, including Paneth (lysozyme-producing cells), goblet (mucin-producing cells), and enteroendocrine cells, as well as absorptive enterocytes [12]. As vitamin D_3_ is easily absorbed by IECs in the small intestine, many studies have focused on the effect of vitamin D_3_ on IEC function [13–15]. The importance of vitamin D_3_ on IEC function has been proven using in vitro or in vivo studies. For example, vitamin D_3_ is known to protect against colorectal cancer by suppressing epithelial cell proliferation and inducing apoptosis [16, 17], while increased apoptotic cell death was observed in the small intestine and colon of vitamin D receptor (VDR)-deficient mice [18]. Furthermore, vitamin D_3_ was found to promote the differentiation of colon carcinoma cells [19]. Indeed, consumption of low levels of dietary vitamin D and calcium in a semi-purified diet or VDR inactivation in leucine-rich repeat-containing G-protein-coupled receptor 5 (Lgr5)-positive ISCs leads to dysfunction of Lgr5^+^ ISCs [20], suggesting that vitamin D_3_ is important for the function of IECs, especially that of ISCs.

Endoplasmic reticulum (ER) stress occurs under homeostatic conditions in the small intestine and is enhanced in intestinal diseases [21]. A variety of stimuli can induce ER stress, including infections, loss of cellular calcium homeostasis, and accumulation of unfolded or misfolded proteins [22]. ER stress subsequently activates the unfolded protein response (UPR), which is necessary for the restoration of normal cell functions, including those of IECs. In general, UPR involves either the survival or apoptotic pathway depending on the ER stress severity [23, 24]. Indeed, UPR is also known to be important for the functions of goblet and Paneth cells, as well as the differentiation of ISCs into transit-amplifying (TA) cells [25, 26]. Therefore, ER stress activation might mediate the effect of vitamin D_3_ on IECs.

Intestinal organoids can mimic ISC proliferation and differentiation forming an intestinal crypt-villus-like structure [27]. Therefore, we utilized intestinal organoid cultures to study the dose-dependent effects of vitamin D_3_ on IEC viability and function, especially in terms of stemness, differentiation, and survival. In addition, we aimed to examine signaling pathways implicated in vitamin D_3_ action.

## Methods

### Preparation of intestinal organoids derived from mouse small intestine

The small intestine was dissected from male C57BL/6N mice (ORIENT Bio, Korea) or LGR-EGFP-IRES-CreERT2 (LGR5-GFP) mice, which were kindly provided by Professor Mi-Na Kweon (College of Medicine/Asan Medical Center, Korea). Intestinal crypts were isolated using the Gentle Cell Dissociation Reagent (StemCell Technologies, MA). The tissue was incubated with 0.1% bovine serum albumin (BSA), and the cell suspension was passed through a 70-μm cell strainer. The isolated crypts were observed under a microscope (CKX53, OLYMPUS, Japan). The crypts were mixed with Matrigel (BD Biosciences, NJ) and Intesticult^TM^ OGM Mouse Basal Medium (StemCell Technologies) at a ratio of 1:1, and 20 μl of the suspended crypts was plated in 48-well plates. After polymerization by incubating at 37 °C for 20 min, 400 μl of Intesticult^TM^ OGM Mouse Basal Medium was added, and the plate was placed in a humidified incubator (5% CO_2_) at 37 °C. The culture medium was replaced every 2 days, and the organoids were passaged every 5 to 6 days.

For passaging, the culture medium was removed, and the organoids were recovered from the Matrigel using the Cell Recovery Solution (Corning, NY). After mechanical disruption by pipetting, the suspended crypts were transferred to microtubes and centrifuged at 850×*g* for 5 min. The crypt pellets were mixed with Matrigel and Intesticult^TM^ OGM Mouse Basal Medium and cultured.

### Vitamin D_3_ treatment

Vitamin D_3_ (1,25-dihydroxyvitamin D_3_) was purchased from Sigma (Sigma-Aldrich, MO) and prepared following the manufacturer’s instructions. Ethanol was used as a vehicle control. Intestinal organoids were treated with various concentrations of vitamin D_3_ (10, 50, and 100 nM), and biological changes were observed after 3 days.

### Endoplasmic reticulum (ER) stress inhibition

Tauroursodeoxycholic acid (TUDCA, MO) was used as a broad ER stress inhibitor. TUDCA was dissolved in dimethyl-sulphoxide (DMSO) according to the manufacturer’s protocol. Intestinal organoids were treated with TUDCA at a concentration of 250 or 500 μM in the presence of vitamin D_3_.

### Assessment of organoid budding

Following treatment with vitamin D_3_ for 3 days, the morphology of intestinal organoids was observed under a light microscope, and the budding was analyzed by measuring the area between expanded organoids from the core using the ImageJ software. The relative value was compared with the non-treated group and presented as the percentage of budding organoids. Data were obtained from four to ten randomly selected fields to acquire ten individual organoids for budding assessment.

### Assessment of organoid viability using MTT reduction

Organoid viability was evaluated using a cell proliferation kit (Roche, Germany). In brief, after culture medium removal, 10 μl MTT-labeling reagent was added to the organoid culture for 1 h. Viable organoids, which could reduce the MTT reagent to formazan, were imaged using a light microscope. After adding DMSO to solubilize the formazan crystals, the absorbance of the colored solution was measured using a microplate reader at 575 nm and a reference at 650 nm. Cell viability was calculated as the percentage of viable cells relative to the non-treated group.

### Detection of apoptosis using the TUNEL assay

The organoids were fixed with 4% paraformaldehyde, and apoptosis was detected using the TUNEL assay kit-BrdU-Red (Abcam, MA) according to the manufacturer’s protocol. Briefly, the fixed organoids were incubated with 70% ethanol for 30 min and then incubated with the DNA-labeling and antibody solutions for 60 and 30 min, respectively. Then, the organoids were stained with DAPI (Sigma-Aldrich) for 1 h and imaged using a confocal microscope (LSM 710; Carl Zeiss) at the Soonchunhyang Biomedical Research Core Facility of the Korea Basic Science Institute.

### Immunofluorescence staining

After medium removal, the organoids were fixed with 4% paraformaldehyde. Permeabilization was performed with 0.2% Triton X-100 followed by a blocking step using 5% BSA. The organoids were incubated with the following primary antibodies: anti-Lgr5 (Abgent, CA), anti-Ki67 (Cell Signaling Technology, MA), anti-Lysozyme (Diagnostic Biosystems, CA), anti-Mucin 2 (Santa Cruz Biotechnology, CA), anti-Chromogranin A (Santa Cruz Biotechnology), anti-Villin (Santa Cruz Biotechnology), and anti-cleaved caspase-3 (Cell Signaling Technology) at 4 °C overnight. Then, the organoids were incubated with the secondary antibodies, either Alexa Fluor 488-conjugated anti-mouse IgG (Life Technologies, MD) or Alexa Fluor 555-conjugated anti-rabbit IgG (Life Technologies), at room temperature for 2 h. The nuclei were stained with DAPI (Sigma-Aldrich) for 1 h, and the organoids were imaged using a confocal microscope (LSM 710; Carl Zeiss). The mean fluorescence intensity was analyzed with the ImageJ software, and the intensity of each marker was normalized to that of DAPI.

### Quantitative real-time polymerase chain reaction (qPCR)

The intestinal organoid culture medium was removed, and the Matrigel dome was washed twice with DPBS. The Matrigel dome was treated with Cell Recovery Solution to completely remove the Matrigel, and total RNA was extracted using Trizol reagent (Ambion, CA). The RNA was converted to cDNA using reverse transcription reagents (TOYOBO, Japan) according to the manufacturer’s protocol. The expression of mRNA was quantified using quantitative polymerase chain reaction with the SYBR Green Real-time PCR Master Mix Kit (TOYOBO). The reaction was performed on a QuantStudio5 Real-Time PCR System (Applied Biosystem^TM^, CA) at the Soonchunhyang Biomedical Research Core Facility of the Korea Basic Science Institute. Target gene expression was calculated by comparing the relative expression levels after normalization to those of *GAPDH*. The primer sequences used are listed in Table 1.

**Table 1:** Primer sequences for qPCR

Primers	Forward sequences (5′ to 3′)	Reverse sequences (5′ to 3′)
*GAPDH*	TTG ATG GCA ACA ATC TCC AC	CGT CCC GTA GAC AAA ATG GT
*Ki67*	CCA GCT GCC TGT AGT GTC AA	TCT TGA GGC TCG CCT TGA TG
*LGR5*	ACC CGC CAG TCT CCT ACA TC	GCA TCT AGG CGC AGG GAT TG
*LYZ*	GAG ACC GAA GCA CCG ACT ATG	CGG TTT TGA CAT TGT GTT CGC
*MUC2*	ATG CCC ACC TCC TCA AAG AC	GTA GTT TCC GTT GGA ACA GTG AA
*CHGA*	AAG GTG ATG AAG TGC GTC CT	GGT GTC GCA GGA TAG AGA GG
*VIL*	GAC GTT TTC ACT GCC AAT ACC A	CCC AAG GCC CTA GTG AAG TCT T
*BRG1*	CAG TGG CTC AAG GCT ATC G	TGT CTC GCT TAC GCT TAC G
*NOTCH1*	AGT GTG ACC CAG ACC TTG TGA	AGT GGC TGG AAA GGG ACT TG
*CHOP*	CTG CCT TTC ACC TTG GAG AC	CGT TTC CTG GGG ATG AGA TA
*ATF6*	CCA ACA GAA AGC CCG CAT T	TGG ACA GCC ATC AGC TGA GA
*tXBP1*	AAG AAC ACG CTT GGG AAT GG	ACT CCC CTT GGC CTC CAC
*sXBP1*	GAG TCC GCA GCA GGT G	GTG TCA GAG TCC ATG GGA

### Statistical analysis

Statistical significance between groups was assessed by the one-way of variance (ANOVA), using the GraphPad software (PRISM 8 Graphpad, CA). A *p*-value of ≤ 0.05 was considered statistically significant. All data presented in each experiment are representative results from three independent biological experiments. Data are presented as mean ± standard deviation, *p ≤ 0.05, **p ≤ 0.01, ***p ≤ 0.0005.

## Results

### Vitamin D_3_ induces small intestine IEC differentiation

**Fig. 1:**
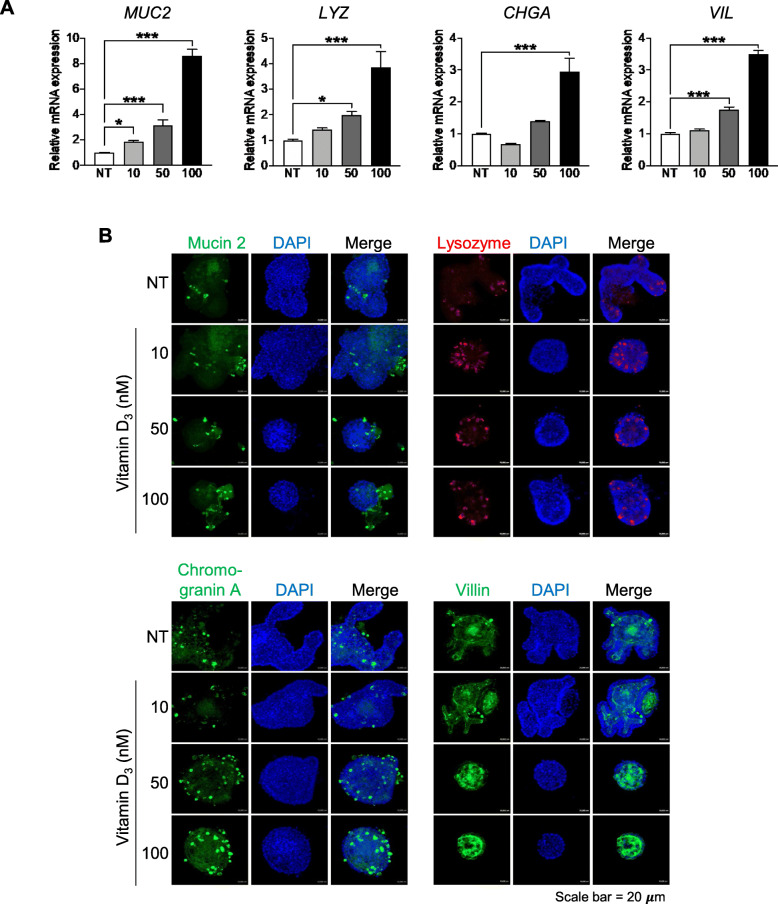
Vitamin D_3_ induces intestinal epithelial cell (IEC) differentiation. Intestinal organoids were treated with various concentrations (10, 50, and 100 nM) of vitamin D_3_, and the expression levels of specific IEC lineage markers, including mucin 2 (*MUC2*), lysozyme (*LYZ*), chromogranin A (*CHGA*), and villin (*VIL*) were quantified using qPCR (**a**). The number of cells in specific IEC lineages were determined using immunofluorescence staining for mucin 2 (goblet cells), lysozyme (Paneth cells), chromogranin A (enteroendocrine cells), and villin (enterocytes) (**b**). Data are presented as mean ± standard deviation, *p ≤ 0.05, ***p ≤ 0.0005

Vitamin D_3_ is known to induce cell differentiation of colorectal cancer-derived epithelial cell lines [7], and epidemiological studies showed that an increasing level of serum vitamin D_3_ is positively correlated with colonic epithelial cell differentiation [13, 28]. However, the direct effect of vitamin D_3_ on small intestine IECs has not been studied. To determine whether vitamin D_3_ influences the differentiation of IECs in the small intestine, we treated small intestinal organoids with various concentrations (10, 50, and 100 nM) of vitamin D_3,_ and the expression levels of specific IEC lineage markers were determined at 3 days post-treatment. The cell lineage markers comprised mucin (*MUC2*), lysozyme (*LYZ*), chromogranin A (*CHGA*), and villin (*VIL*), which are markers for goblet, Paneth, enteroendocrine cells and enterocytes, respectively. The expression level of *MUC2* was significantly increased by vitamin D_3_ at all treated concentrations (approximately 2- to 10-fold compared with that of the non-treated group). The expression levels of *LYZ* and *VIL* were significantly increased by 50 or 100 nM vitamin D_3_ (approximately 2- to 4-fold compared with those of the non-treated group). Moreover, the expression level of *CHGA* was significantly upregulated by 100 nM vitamin D_3_ (3-fold compared with that of the non-treated group) (Fig. 1a). To confirm the vitamin D_3_-induced increase in IEC differentiation, the numbers of specific differentiated IECs were examined using immunofluorescence staining. Consistent with the gene expression levels, the numbers of each differentiated IEC type increased with vitamin D_3_ treatment, especially by treatment with 100 nM vitamin D_3_ (Fig. 1b). The increased numbers of differentiated IECs were confirmed by the mean fluorescence intensities of all lineage markers, as presented in Figure S1A. The mean fluorescence intensities of Mucin 2 and Villin were significantly increased by treatment with 100 nM vitamin D_3_, while the intensities of lysozyme and chromogranin A were significantly elevated by treatment with both 50 and 100 nM vitamin D_3_. Taken together, these data suggest that vitamin D_3_ induces general IEC differentiation in the small intestine.

### Vitamin D_3_ suppresses IEC stemness and proliferation

**Fig. 2:**
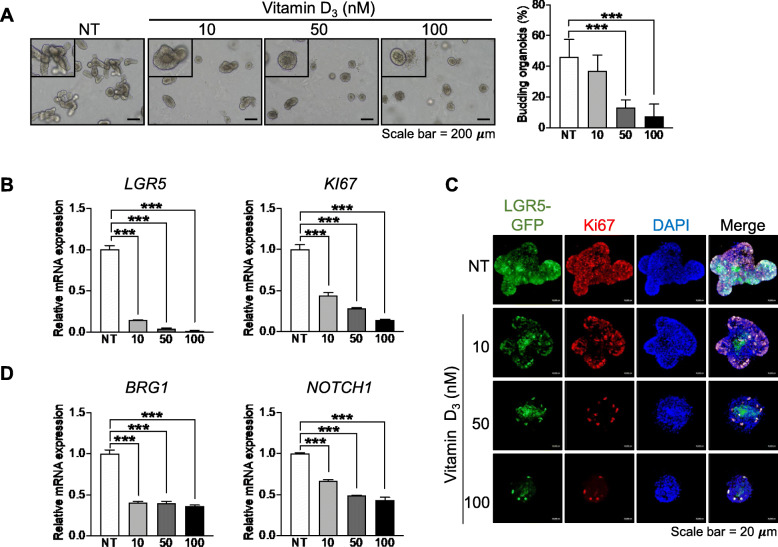
Vitamin D_3_ reduces budding and suppresses cell proliferation and stemness. Intestinal organoids derived from the murine small intestine were treated with various concentrations (10, 50, and 100 nM) of vitamin D_3._ The formation of intestinal organoids was observed with a light microscope (left panel, magnification, ×100), and the percentage of budding organoids was analyzed using the ImageJ software (right panel) (**a**). The expression levels of leucine-rich repeat-containing G-protein-coupled receptor 5 (*LGR5*) and *Ki67*, markers of ISCs and cell proliferation, respectively, were quantified using qPCR (**b**). Intestinal organoids derived from LGR5-green fluorescent protein (GFP) mice were treated with vitamin D_3._ The number of LGR5-GFP-positive cells was visualized using a confocal microscope (green fluorescence), and the number of Ki67-positive cells was determined following immunofluorescence staining (red fluorescence) (**c**). The expression levels of Brahma-related gene 1 (*BRG1)* and *NOTCH 1* were quantified using qPCR (**d**). Data are presented as mean ± standard deviation, ***p ≤ 0.0005

The effect of vitamin D_3_ on IEC proliferation and stemness in the small intestine under normal conditions remains to be determined. To study the effect of vitamin D_3_ on ISCs, small intestinal organoids were treated with various concentrations (10, 50, and 100 nM) of vitamin D_3,_ and the number of budding organoids was determined, as the crypt-villus formation of intestinal organoids originates from the renewal and proliferation of ISCs [27]. We found that the percentage of budding organoids was significantly reduced by 50 or 100 nM vitamin D_3_ (Fig. 2a), suggesting the malfunction of ISCs upon vitamin D_3_ treatment. To further determine whether the reduction in budding was caused by the depletion of ISCs or cell proliferation, the expression levels of *LGR5* and *Ki67* (markers for ISCs and cell proliferation, respectively) were quantified using qPCR. The expression levels of both *LGR5* and *Ki67* were dramatically downregulated by vitamin D_3_ at all concentrations tested (Fig. 2b). Next, the intestinal organoids derived from LGR5-GFP-positive cells were utilized to confirm the depletion of ISCs and cell proliferation. After treatment with vitamin D_3_, the LGR5-GFP signal was observed under a fluorescence microscope, and Ki67-positive cells were evaluated using immunofluorescence staining. Consistent with the gene expression levels, the LGR5-GFP signal was negatively correlated with the vitamin D_3_ concentration. Moreover, the number of Ki67-positive cells was also decreased by vitamin D_3_ treatment, especially by 50 or 100 nM of vitamin D_3_ (Fig. 2c and Figure S1B), suggesting that vitamin D_3_ suppresses IEC stemness and cell proliferation.

Notch signaling is known to regulate ISC function in the adult small intestine [29]. Furthermore, a previous study showed that the transcription activator brahma-related gene 1 (BRG1) plays a role in intestinal growth, crypt-villus formation, and stemness through the regulation of Notch1 signaling [30]. As our data showed that vitamin D_3_ could suppress ISCs, we also quantified the expression of *BRG1* and *NOTCH1* in vitamin D_3_-treated organoids. We found that the expression levels of both *BRG1* and *NOTCH1* were significantly decreased by vitamin D_3_ treatment (Fig. 2d). Taken together, these data suggest that vitamin D_3_ causes ISC depletion and inhibits IEC proliferation in the small intestine.

### Vitamin D_3_ promotes apoptotic cell death in intestinal organoids

**Fig. 3:**
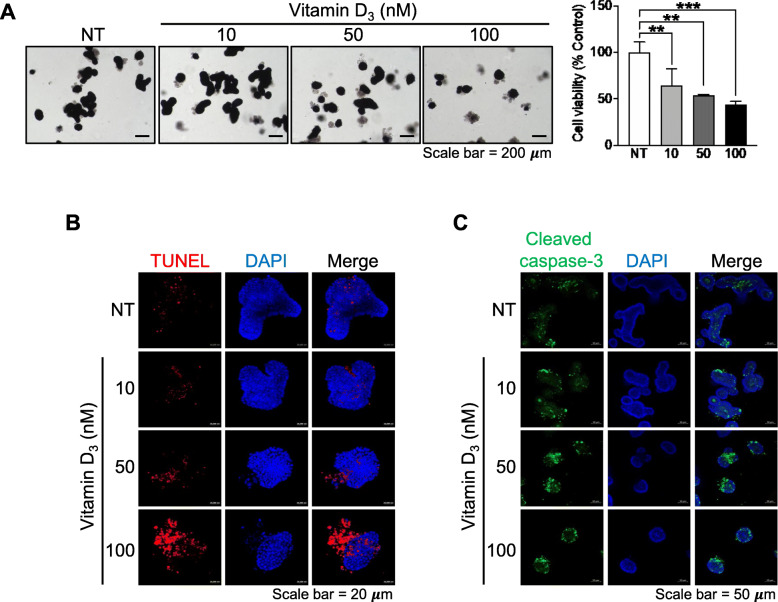
Vitamin D_3_ promotes apoptotic cell death. Intestinal organoids were treated with various concentrations (10, 50, and 100 nM) of vitamin D_3_. The percentage of viability was evaluated by the reduction of the MTT reagent (**a**). Apoptotic cells were identified using the TUNEL assay (red fluorescence) (**b**) and immunofluorescence staining for cleaved caspase-3 (green fluorescence) (**c**). Data are presented as mean ± standard deviation, **p ≤ 0.01, ***p ≤ 0.0005

Previous studies showed that vitamin D_3_ is associated with IEC survival [7, 18]. However, there is no evidence showing the direct effect of vitamin D_3_ on IECs in the small intestine, where vitamin D_3_ is readily absorbed. As our results showed that vitamin D_3_ could suppress IEC stemness and proliferation, we hypothesized that vitamin D_3_ might alter intestinal viability due to the depletion of ISCs. Intestinal organoids were treated with various concentrations of vitamin D_3_, and organoid viability was assessed using the MTT assay. Viable cells reduced MTT to formazan, which is represented by the dark purple color in the organoids. The number of organoids containing formazan was decreased by vitamin D_3_ treatment at all tested concentrations (Fig. 3a), suggesting that vitamin D_3_ reduces IEC viability. As the association between apoptotic IEC death and VDR was previously described [18], we hypothesized that vitamin D_3-_induced reduction in organoid viability might be mediated through apoptosis. Intestinal organoid apoptosis was determined using the TUNEL assay and immunofluorescence staining for cleaved caspase-3. The results showed that the signal was dramatically increased following 50 or 100 nM vitamin D_3_ treatment in intestinal organoids treated with the TUNEL reagents (Fig. 3b). In addition, the immunofluorescence signal of cleaved caspase-3 was also increased following 50 or 100 nM vitamin D_3_ treatment (Fig. 3c and Figure S1C). Taken together, these data confirm that high levels of vitamin D_3_ promote apoptotic cell death in the small intestine.

### Vitamin D_3_ suppresses stemness by augmenting ER stress

**Fig. 4:**
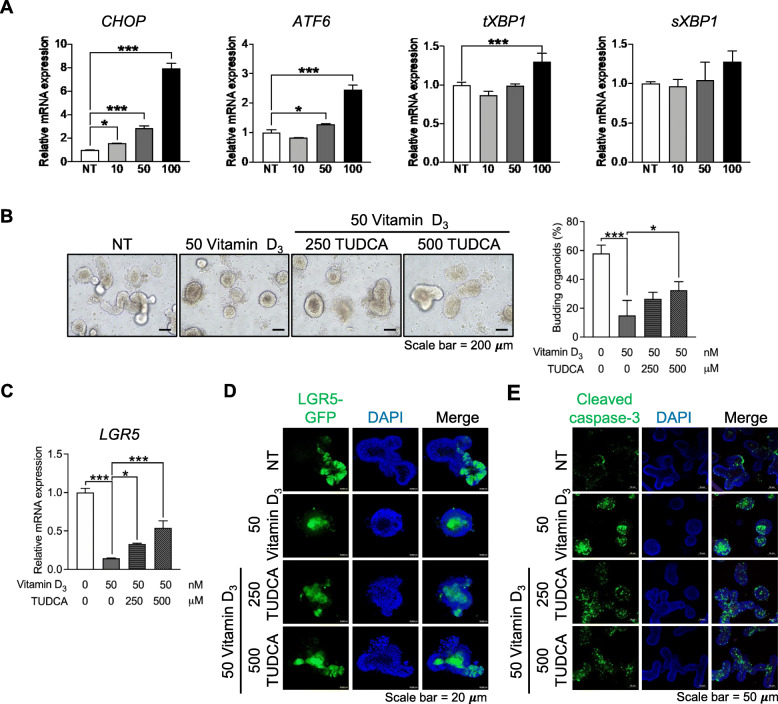
The effect of vitamin D_3_ on small intestinal organoids is mediated by endoplasmic reticulum (ER) stress induction. Intestinal organoids were treated with various concentrations (10, 50, and 100 nM) of vitamin D_3_ and the expression levels of unfolded protein response (UPR) genes, C/EBP homologous protein (*CHOP)*, activating transcription factor 6 (*ATF6*), total form X-box-binding protein 1 (*tXBP1*), spliced form (*s*)*XBP1* were quantified using qPCR (**a**). Small intestinal organoids derived from leucine-rich repeat-containing G-protein-coupled receptor 5-green fluorescent protein (LGR5-GFP) mice were treated with 50 nM vitamin D_3_ in the presence or absence of the ER stress inhibitor tauroursodeoxycholic acid (TUDCA) (250 or 500 nM). The budding of intestinal organoids was observed under a microscope (left panel), and the percentage of budding organoids was analyzed using the ImageJ software (right panel) (**b**). The expression level of *LGR5* was quantified using qPCR (**c**), and the numbers of LGR5-GFP-positive cells were visualized using a confocal microscope (green fluorescence) (**d**). Immunofluorescence images of cleaved caspase-3 (green fluorescence) (**e**). Data are presented as mean ± standard deviation, *p ≤ 0.05, ***p ≤ 0.0005

ER stress augmentation can lead to ISC loss and induce IEC differentiation by UPR activation [25]. Furthermore, UPR is involved in cell survival [23, 24]. As stemness and viability were reduced in vitamin D_3_-treated organoids, we hypothesized that the action of vitamin D_3_ on stemness and viability might be mediated by ER stress induction. First, we determined whether ER stress is induced by vitamin D_3_ treatment. The expression levels of UPR genes, the C/EBP homologous protein (*CHOP*), the activating transcription factor 6 (*ATF6*), and the X-box-binding protein 1 (*XBP1*) in either total form (*tXBP1*) or spliced form (*sXBP1*) were quantified using qPCR. The results showed that the expression level of *CHOP* was significantly increased by vitamin D_3_ at all tested concentrations. The expression level of *ATF6* was significantly upregulated by vitamin D_3_ at 50 or 100 nM. In addition, the expression level of tXBP1 was significantly increased by 100 nM vitamin D_3_; however, the expression of *sXBP1* did not change with vitamin D_3_ treatment (Fig. 4a), suggesting that D_3_ treatment induces ER stress.

Secondly, we determined whether ER stress induction mediates the action of vitamin D_3_ on IEC stemness and apoptotic cell death. According to our results, 50 nM vitamin D_3_ is the minimum concentration that induces UPR gene expression, reduces stemness, and promotes apoptotic cell death. Therefore, intestinal organoids were treated with 50 nM vitamin D_3_ in the presence or absence of 250 μM or 500 μM TUDCA, a classical ER stress inhibitor, and budding was observed. The results showed that the percentage of budding was significantly rescued in the presence of 500 μM TUDCA (Fig. 4b), which might be a result of stemness induction. To confirm that ER stress inhibition could rescue IEC stemness, the expression level of *LGR5* was quantified using qPCR, and the signal from GFP was visualized using a confocal microscope. We found that the expression level of *LGR5* was significantly upregulated in the presence of TUDCA (Fig. 4c). In addition, the mean fluorescence intensity of LGR5-GFP was partially rescued by TUDCA treatment (Fig. 4d and Figure S2A). Lastly, immunofluorescence staining of cleaved caspase-3 showed that vitamin D_3_ treatment in the presence of TUDCA could reduce the cleaved caspase-3 signal compared with that of vitamin D_3_ treatment alone (Fig. 4e and Figure S4B), suggesting that the effect of vitamin D_3_ on apoptotic cell death is mediated by ER stress induction. Furthermore, we did not observe changes in *Ki67*, *LYZ*, and *MUC2* expression levels, while the expression levels of *CHGA* and *VIL* were significantly increased in the presence of 250 or 500 μM, and 500 μM TUDCA, respectively (Figure S2C). Thus, our findings suggest that vitamin D_3_ suppresses IEC stemness and promotes apoptotic cell death partially through ER stress activation.

## Discussion

Vitamin D_3_ plays an important role in many biological processes, such as intestinal calcium absorption, maintenance of intestinal epithelial integrity and function, and cancer suppression [3, 7, 8]. However, the levels of vitamin D_3_ in the body should be well regulated to avoid side effects. For example, vitamin D_3_ deficiency could lead to disease development, such as inflammatory bowel disease and pancreatitis [9]. In contrast, excess vitamin D_3_ levels could alter the intestinal microbiota composition and increase disease susceptibility [10]. Most studies of vitamin D_3_ effects on IECs have been conducted using colorectal cancer cell lines and in vivo mouse models, specifically under disease induction, such as colitis, mainly focusing on the colon [7, 13, 20]. As vitamin D_3_ is mainly absorbed in the small intestine, the effect of vitamin D_3_ on IECs in the small intestine should be understood.

Many IEC cell lineages are generated from ISCs, including secretory cells, such as Paneth (lysozyme-producing cells), goblet (mucin-producing cells), and enteroendocrine cells, as well as absorptive enterocytes [12]. ISCs undergo proliferation and differentiation for homeostatic turnover of the intestinal epithelium and ensure epithelial regeneration following intestinal damage [31, 32]. Previous studies showed that vitamin D_3_ influences the proliferation and differentiation [7, 33] as well as the survival of IECs in the colon [34]. Therefore, we hypothesized that vitamin D_3_ might also affect the differentiation, proliferation, stemness, and survival of IECs in the small intestine under normal conditions. We used intestinal organoids to assess the effect of vitamin D_3_ on IECs in the small intestine. Consistent with the effects of vitamin D_3_ on colonic IECs [7, 18], we demonstrated that vitamin D_3_ globally induced IEC differentiation into specific cell lineages, including goblet, Paneth, enteroendocrine cells, and enterocytes, represented by the increased expression of mucin-2, lysozyme, chromogranin A, and villin, respectively. It is well known that cell differentiation and proliferation are coordinately regulated by the growth factors present in the microenvironment, including those in the small intestine [35]. While vitamin D_3_ increased cell differentiation, it drastically suppressed IEC proliferation, represented by the reduction in Ki67-positive cells in intestinal organoids. As ISCs are highly proliferative cells [36], we hypothesized that the decrease in IEC proliferation might be due to the suppression of ISCs. Unlike normal colon organoids derived from humans, where vitamin D_3_ upregulates stem cell-related genes [33], our data showed that vitamin D_3_ inhibited the expression level of *LGR5* and decreased the number of LGR5-GFP-positive cells in small intestinal organoids derived from LGR5-GFP mice, suggesting that vitamin D_3_ reduces stemness in the small intestine. Moreover, we revealed the downregulation of the gene expression levels of *BRG1* and *Notch1*, which are also known to regulate ISC function and IEC differentiation [29, 30], as well as the reduction of budding organoids, which support the effect of vitamin D_3_ on ISC depletion. However, direct evidence showing that vitamin D_3_ either influences stemness maintenance or proliferation needs to be demonstrated in further studies. The discrepancy between the effects of vitamin D_3_ on the organoids derived from the small intestine and colon might be explained by several possibilities, including the components in the crypt base compartment, the proliferative rate as well as the distinct molecular signature, and intrinsic regulation in stem cell population [37, 38]. Notably, vitamin D_3_ concentrations might be important for the effect of vitamin D_3_ on IECs in the small intestine. Previous studies showed that vitamin D_3_ can reduce the viability of colorectal carcinoma cell lines via the induction of apoptosis in a dose-dependent manner [16, 17]; therefore, we hypothesized that the depletion of intestinal stemness by vitamin D_3_ may be associated with apoptosis-induced cell death. Our results showed that vitamin D_3_ promotes apoptotic cell death, especially at high doses. In addition, our study may support a potential biological significance of vitamin D_3_ in cancer stem cell therapy, which has been reviewed previously [39, 40]. Vitamin D_3_ may have a probable beneficial role in the inhibition of progression and survival, as well as in facilitating the apoptosis of cancer stem cells, resulting in reduction of the self-renewal capacity that initiates tumor formation. However, whether apoptotic cell death mainly occurs in ISCs, proliferative cells, or differentiated cells remains to be determined. Therefore, the specific IEC lineage targeted by vitamin D_3_ needs to be identified.

ER stress induction is known to be related with cell survival or apoptotic cell death, depending on the severity of ER stress [23, 24]. Furthermore, the activation of UPR upon ER stress induction is associated with the functions of goblet and Paneth cells and is important for the differentiation of ISCs into TA cells [25, 26]. Therefore, we hypothesized that the effect of vitamin D_3_ on IEC stemness and survival in the small intestine might be mediated by ER stress induction. Our results showed that the expression levels of UPR genes, especially those of C/EBP homologous protein (CHOP) and activating transcription factor 6 (ATF6), were upregulated in vitamin D_3_-treated organoids. However, treatment with TUDCA, which is known to reduce experimental colitis by abolishing ER stress in colonocytes [41], partially rescued the vitamin D_3_-induced depletion of LGR5-GFP-positive cells and reduced the number of cleaved caspase-3-positive cells in intestinal organoids, suggesting that the depletion of stemness and apoptotic cell death induction by vitamin D_3_ may be mediated by ER stress induction. Although our findings showed that ER stress is partially involved in the depletion of stemness and induction of apoptotic cell death, additional mechanisms are likely involved, which remain to be identified. Our study provides evidence that vitamin D_3_ alters the proliferation, differentiation, stemness, and survival of IECs in the small intestine. Moreover, the effect of vitamin D_3_ on IEC stemness and survival is partially mediated by ER stress induction.

## Conclusions

In summary, this study revealed that vitamin D_3_ could induce cell differentiation, promote apoptotic cell death, and suppress cell proliferation and stemness in the small intestine partially through the activation of ER stress. These effects are similar to those of drugs that regulate cell survival via the activation of ER stress [42]. Future studies are required to determine the detailed mechanism underlying the regulation of IEC function by vitamin D_3_, specifically that of ISCs, which is important for controlling the levels of vitamin D_3_ and maintaining intestinal homeostasis.

## Supplementary Information

**Additional file 1.**

## Data Availability

The datasets used and/or analyzed during the current study are available from the corresponding author on reasonable request.

## Abbreviations

IECs: Intestinal epithelial cells
ER: Endoplasmic reticulum
LGR5: Leucine-rich repeat-containing G-protein-coupled receptor 5
CHOP: C/EBP homologous protein
ATF6: Activating transcription factor 6
TUDCA: Tauroursodeoxycholic acid
VDR: Vitamin D receptor
ISCs: Intestinal stem cells
UPR: Unfolded protein response
TA: Transit-amplifying
MUC2: Mucin 2
LYZ: Lysozyme
CHGA: Chromogranin A
VIL: Villin
GFP: Green fluorescent protein
BRG1: Brahma-related gene 1
XBP1: X-box-binding protein 1
