# Supplementary material for: Vitamin D3 suppresses intestinal epithelial stemness via ER stress induction in intestinal organoids
Source: Stem Cell Res Ther. 2021 May 13;12:285. doi: 10.1186/s13287-021-02361-2 (PMC8117327; doi:10.1186/s13287-021-02361-2)
Supplement: Supplementary file 1 — Additional file 1. [file 13287_2021_2361_MOESM1_ESM.pdf]

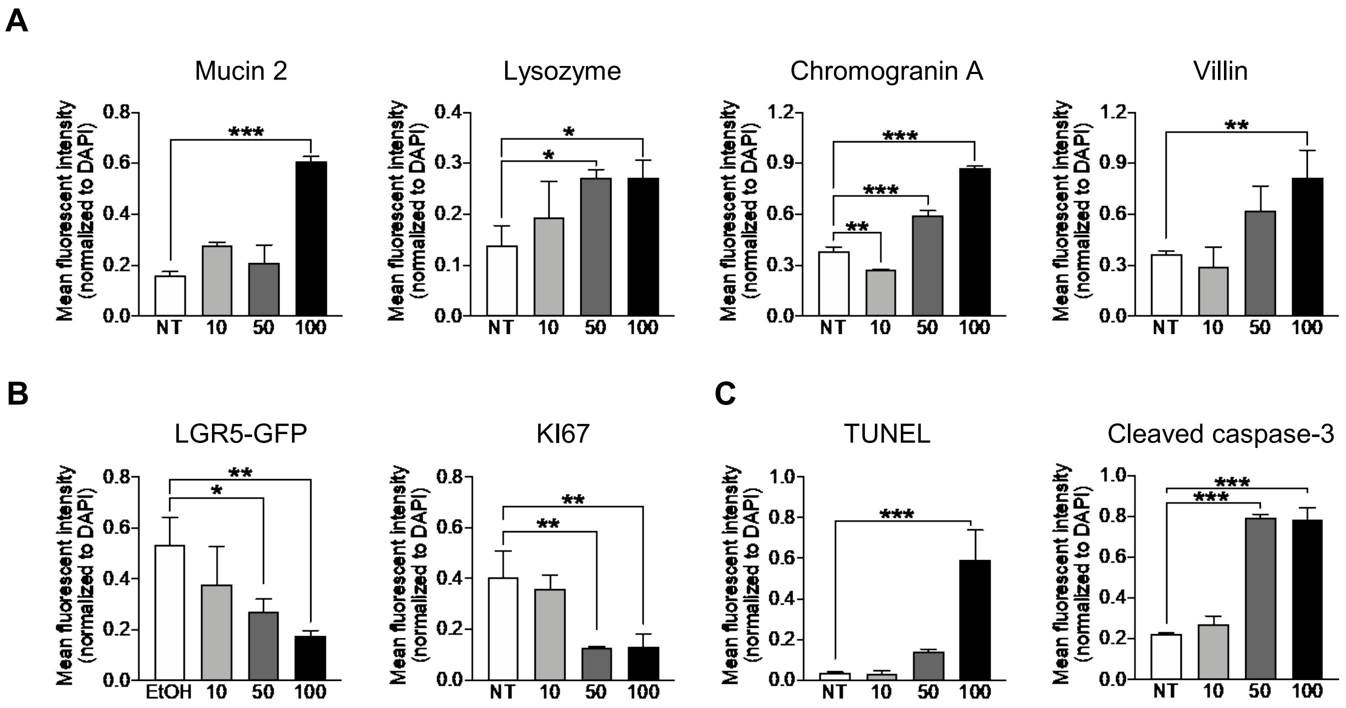

Figure S1: Quantitative assessment of mean fluorescence intensity. The mean fluorescence intensity was analyzed using the ImageJ software and normalized to that of DAPI. Intestinal epithelial cell lineage markers (Mucin 2, Lysozyme, Chromogranin A, and Villin) **(A)**, stem cell marker (LGR5-GFP), proliferative marker (KI67) **(B)**, and cell apoptotic markers (TUNEL and Cleaved caspase 3) **(C)**. Data are presented as mean ± standard deviation, *$p \leq 0.05$, **$p \leq 0.01$, ***$p \leq 0.0005$.

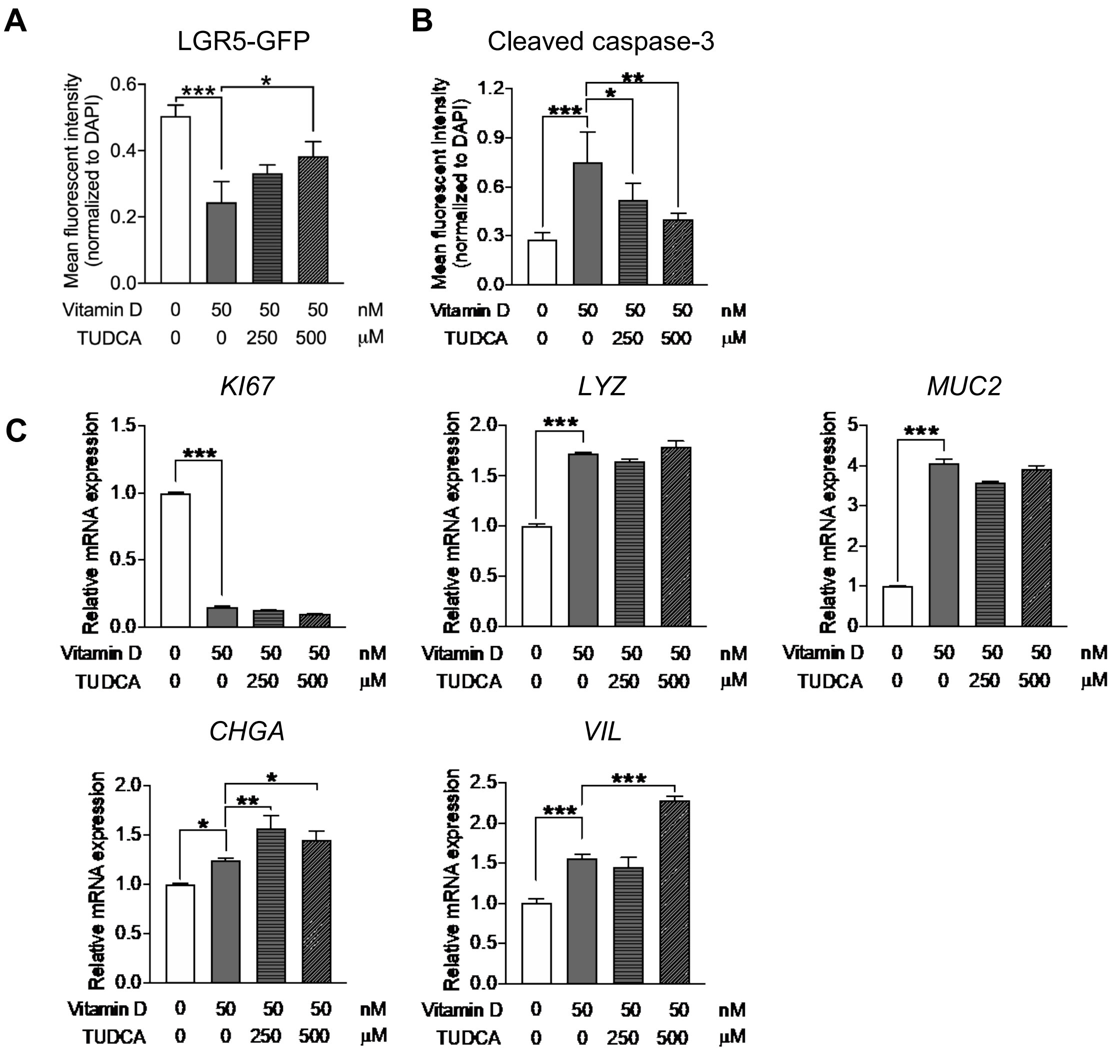

Figure S2: ER stress inhibition may suppress intestinal apoptosis and induce stemness, as well as differentiation into enteroendocrine cells and enterocytes. Intestinal organoids were treated with 50 nM vitamin D_3 in the presence or absence of 250 μM or 500 μM TUDCA. The mean fluorescence intensity of LGR5-GFP **(A)** and cleaved caspase-3 **(B)** was analyzed with the ImageJ software and normalized to DAPI. The expression levels of *KI67*, *LYZ*, *MUC2*, *CHGA*, and *VIL* were quantified with qPCR **(C)**. Data are presented as mean ± standard deviation, $^{*}p \leq 0.05$, $^{**}p \leq 0.01$, $^{***}p \leq 0.0005$.

Figure S2
